# Assessment of transfer-time and time-to-surgery as risk factors to survival in Gastroschisis (GS) in a LMIC; an eight-year review

**DOI:** 10.1007/s00383-024-05872-0

**Published:** 2024-11-07

**Authors:** Alaa Obeida, Rawan El-Hussein, Hadeer Mohamed NasrEldin, Mohammad Allam, Khaled Bahaaeldin, Sherif Kaddah, Aly Shalaby

**Affiliations:** Department of Paediatric Surgery, Kasr AlAiny Faculty of Medicine, Cairo University Specialized Paediatric Hospital, Cairo University, Ali Ibrahim Street, Mounira, Cairo, 11241 Egypt

**Keywords:** Gastroschisis, Abdominal wall defects, Transfer time, Africa, Egypt

## Abstract

**Background:**

The management of Gastroschisis in LMICs continues to be a challenge and is associated with very poor outcomes in contrast with HICs where survival rates near 100%. The purpose of this work is to provide an overview of survival over the past 8 years in a high-flow tertiary centre in Africa. It also investigates the effect of transfer-time and time-to-surgery on outcome.

**Methods:**

Retrospective case note review of all GS admissions. The variables assessed were gender, gestational age, weight, type of GS, transfer time, time to surgery and type of surgery. The primary outcome was survival.

**Results:**

A total of 171 GS cases were identified: 148 simple, 23 complex. Seven died before surgery. The median age at surgical intervention was 8.5 h (range, 0–48). Closure options ranged from single-staged (primary fascial, skin, umbilical flap and sutureless closure) or a staged (silo) closure. Overall survival was 34.5%. Cases transferred under 8 h had a 46% survival. Surgery under 12 h of life had highest survival, 45%. Simple GS survived better than complex GS (40% vs 10%). Primary closure had a significantly better survival compared to staged closure (51% vs 18%).

**Conclusions:**

Transfer-time < 8 h plays a vital role in survival of GS cases. Surgical intervention within 12 h of birth showed a statistically significant improvement in outcome. Primary closure was associated with better survival rates.

**Level of Evidence:**

Level III.

## Introduction

Gastroschisis (GS) is a congenital anterior abdominal wall defect characterized by herniation of intra-abdominal viscera into the amniotic cavity without membrane cover [1, 2]. The cause of GS is unknown in most newborns [3]. Its overall prevalence is 3.06 per 10,000 live births and has shown an increasing incidence in many countries [4]. Although the annual incidence of most congenital anomalies is constant across geographies, GS shows an increased annual incidence in several regions/countries worldwide [5–7].

Most cases are classified as simple GS and have a low morbidity with a high survival rate in high-income countries (HICs). Approximately 11–17% of patients are categorized as complex with an associated atresia, stenosis, perforation, necrosis, or volvulus. These complex patients are at greater risk of prolonged hospital stay, short gut syndrome, and mortality [8, 9].

Sub- Saharan Africa, where up to 50% of the population are children, has almost one-third of the worlds burden of surgical disease and half of the world’s under-5 deaths [10–12]. A population-based study in Uganda highlighted that almost one-third of childhood deaths resulted from surgical conditions [13].

GS mortality has decreased in the past 60 years in both HICs and middle-income countries (MICs) countries but not in low-income countries (LICs) [14]. HICs have GS mortality rates below 10% [14], while (MICs) have intermediate results, and LICs may have mortality rates ranging from 75 to 100% [15].

Many families in LMICs travel several hours at great personal expense to reach a healthcare facility [16]. Unfortunately, many unequipped healthcare facilities cannot treat paediatric surgical cases, such as GS, so patients and their families get referred to tertiary-level hospitals for care [17, 18]. When this happens, infants with GS are at increased risk of dying due to a lack of timely treatment [19, 20].

The purpose of this study is to present a cumulative look at the GS experience of a tertiary paediatric surgery centre in Egypt with a focus on assessing the value of transfer-time and time-to-surgery on cases’ survival.

## Methods

This is a retrospective cohort study including all infants born with GS presenting to the Cairo University Specialized Paediatric Hospital (CUSPH), Surgical Neonatal Intensive Care Unit (SNICU) over a period of eight years from January 2016 till January 2024.

Data were obtained from the SNICU database after taking the necessary ethical approval from the ethical committee at Cairo University Hospitals. Data retrieved were anonymised and divided into patient and treatment variables. Patient variables included gender, gestational age, gestational weight, and transfer-time. Transfer time was defined as the time in hours from birth till reaching the tertiary centre to receive treatment. Time to surgery was defined as the time in hours from birth to start of surgical intervention. Treatment variables included complexity of GS, age at surgical intervention in hours, type of surgical closure, need for re-do surgery and outcome. Patient’s outcome was recorded as live discharge or death.

A standardized management protocol is followed for the GS cases presented to our unit since October 2017 [21, 22]. The pre-operative management protocol follows the following steps: The baby is positioned lengthwise on a resuscitaire or warmer to facilitate access. ECG lead, probes for temperature and oxygen saturation are connected. Kinking of the bowel is avoided by laying the child on their side or by propping up the bowel with gauze rolls in the supine position. Bowel loops are covered with a sterile cling film if not already performed in transfer, nasogastric tube insertion for decompression, urinary catheter insertion and fluid resuscitation. Resuscitation follows APLS guidelines of airway, breathing, circulation, rapid initial examination. Bloods are taken for blood sugar (if not done earlier), a complete blood picture, kidney and liver functions, clotting and a cross-match. Broad-spectrum antibiotics are given according to the hospital protocol [22]. Surgical closure was done in a sterile environment either bedside or in theatre. All silos were surgically-formed using a malleable sterilized plastic sheet.

Descriptive analysis tools that were used for single variables are median and range. Analytical tests were done using chi-square and Fisher’s exact tests to correlate different variables with the outcome. Significance was accorded at a P value < 0.05.

## Results

A total of 171 patients with GS were admitted to the SNICU during the eight-year period, representing around 6.1% of neonatal surgical admissions on the unit.

Females represented 50.3% (86/171) of the cohort. The median gestational age was 38 weeks (range, 27–43). The mean birth weight was 2.3kg (range, 1–3.7).

Our centre is a standalone paediatric hospital with no maternity section. Therefore all 171 cases were out-born. Seven cases died before surgery giving a denominator of 164 cases for analysis of post-operative outcomes. Five cases were excluded from transfer time calculation due to lack of documentation giving a denominator of 166 cases for analysis of transfer time. The median transfer time to the SNICU was 5.5 h (range, 0–36).

Surgery took place after admission and resuscitation. Eleven cases did not have a documented time to surgery, leaving 153 cases for analysis. Of these, 93 (60.8%) neonates underwent surgery in less than 12 h, 47 (30.7%) were operated on between 12–24 h and 13 (8.5%) after 24 h. The median age at surgical intervention was 8.5 h (range, 0–48).

The cohort was composed of simple (n = 148) and complex (n = 23) GS cases. Seven (4%) neonates died before any surgical intervention.

**Fig. 1:**
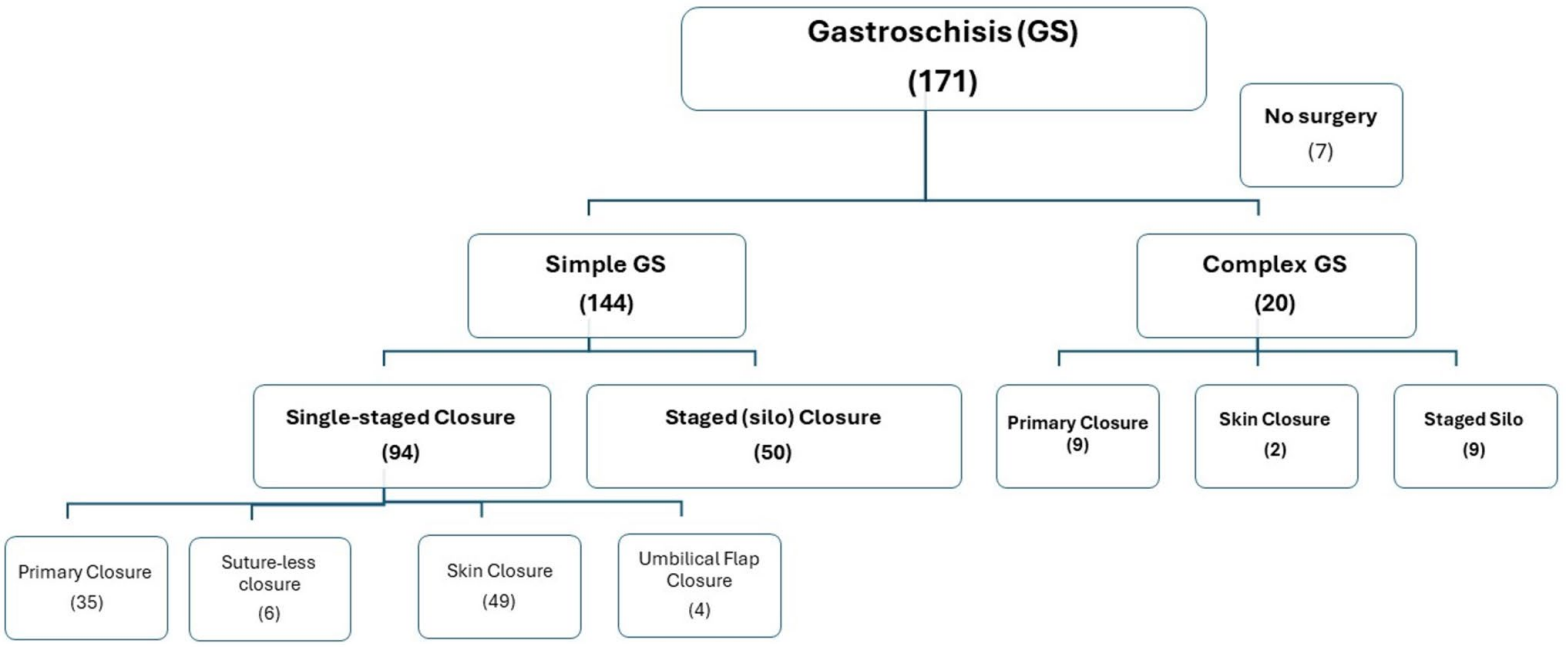
Classification of the GS cohort according to surgery

Simple GS (n = 144, 87.8%) had either a single-staged closure (n = 94/144, 65%) or a staged (silo) closure (n = 50, 35%). Surgical options in the single-staged group were primary fascial closure (n = 35/94, 37%), skin closure (n = 49, 52%), umbilical flap closure (n = 4, 4%) and sutureless closure (n = 6, 6%) **(**Fig. 1**)**.

Complex GS (n = 20/164, 12.2%) surgical repairs included primary fascial closure (n = 9/20, 45%), staged surgical silo (n = 9, 45%), and skin closure (n = 2, 10%) **(**Fig. 1**)**.

Twenty cases (20 cases, 12%) developed post-operative complications and had redo surgery which included redo silo (n = 9/20, 45%), stoma formation (n = 5, 25%), adhesiolysis (n = 3, 15%), resection and anastomosis (n = 2, 10%), and one case (5%) had a burst abdomen post-primary closure.

**Fig. 2:**
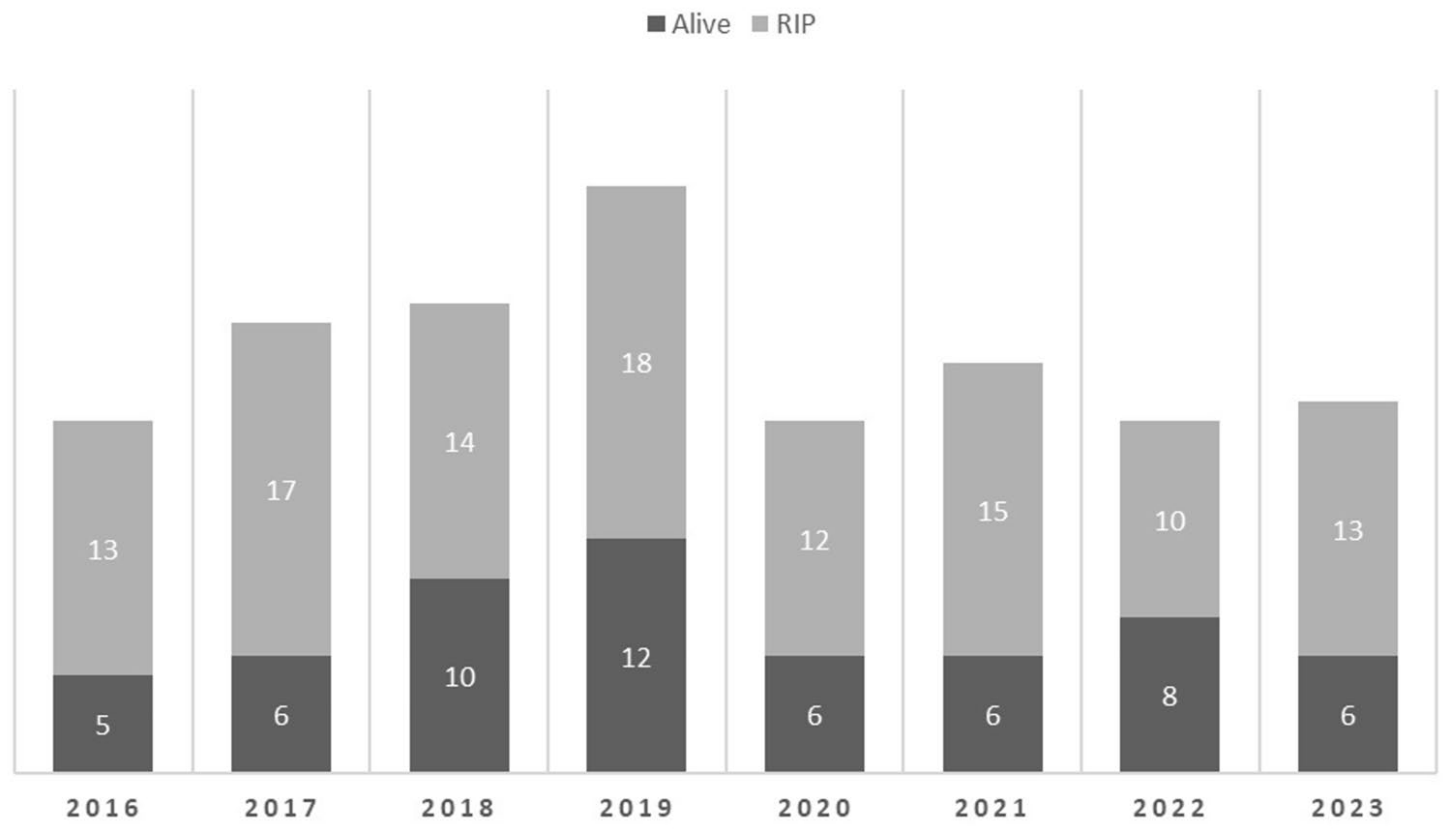
Change of GS survival over years (2016–2023)

The survival rate over the 8 years was 34.5% (59 /171) and ranged from 26 to 44%. The survival trend is shown in the graph (Fig. 2).

There was no difference between female and male survival (37.2% vs 31.8%). Full-term neonates had a higher survival rate than pre-term ones (41% vs 21%), P = 0.02. Cases with a normal birth weight (> 2.5 kg) showed a statistically significant survival compared to their low birth weight and very low birth weight counterparts (55.6% vs 22.8% vs 33.3% respectively), P = 0.0002.

**Fig. 3:**
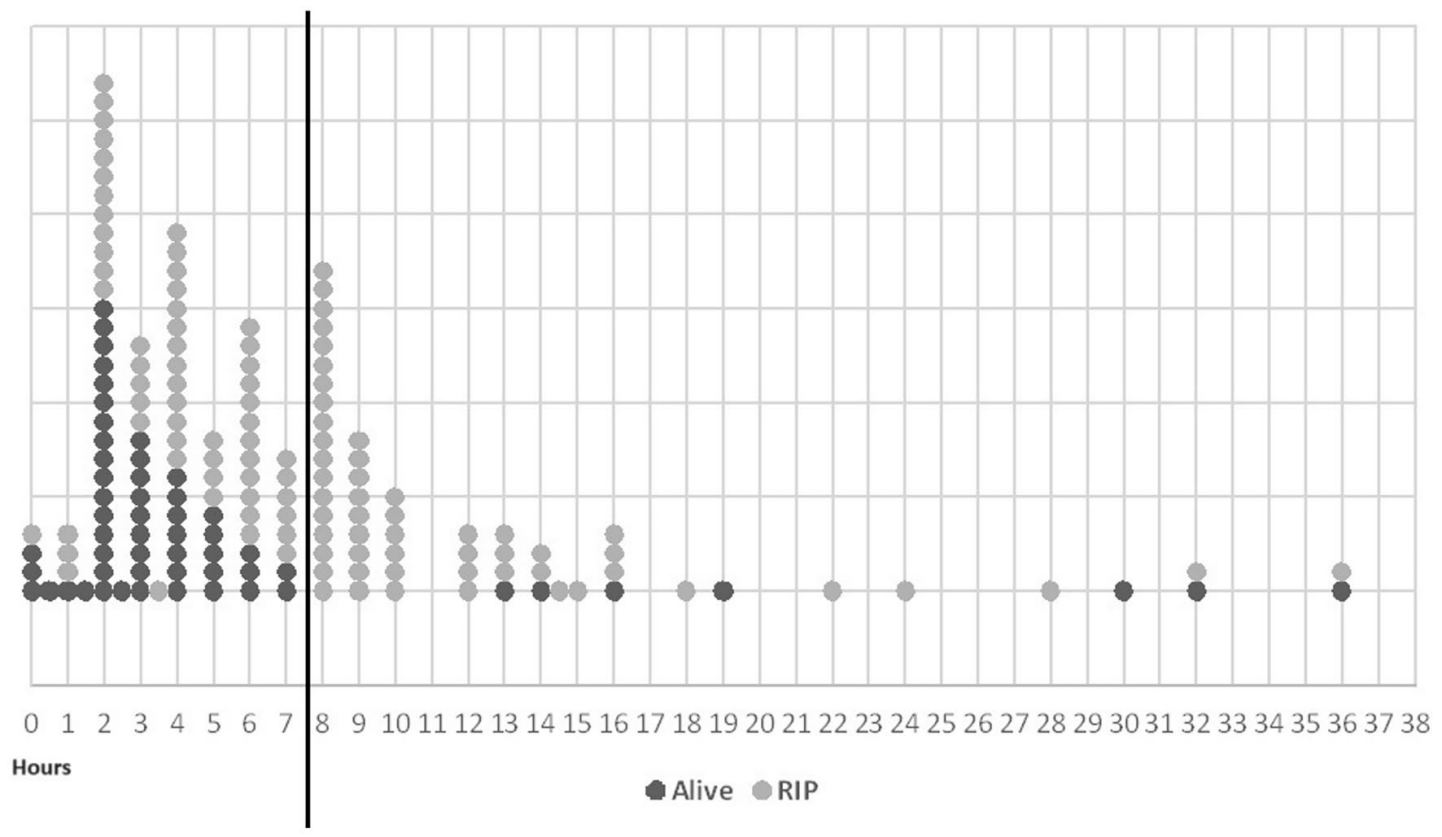
GS transfer time in hours/survival

A transfer time under 8 h (n = 106/166, 64%) resulted in a 46% (n = 49) survival rate compared to an 11.7% (n = 7) survival rate for cases transferred at or after 8 h of life (n = 60/166, 36%). P = 0.000002 **(**Fig. 3**)**.

Surgical intervention under 12 h after birth (n = 93/153) resulted in the highest survival (n = 42/93, 45%) compared to either surgery between 12 to 24 h (n = 10/47, 21%), or surgery longer than 24 h (n = 2/13, 15%), P = 0.005.

**Fig. 4:**
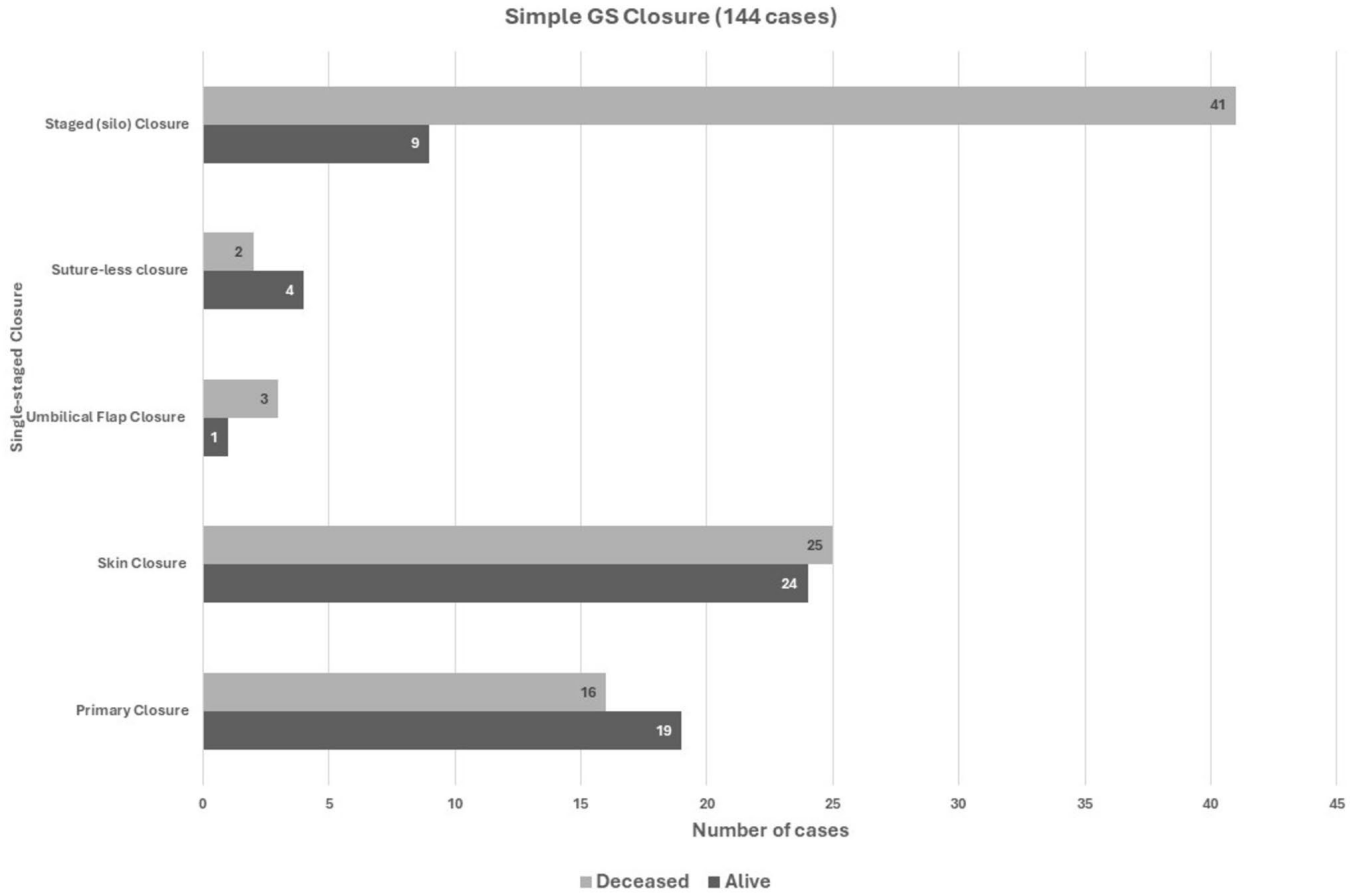
Surgical outcome in Simple GS

**Fig. 5:**
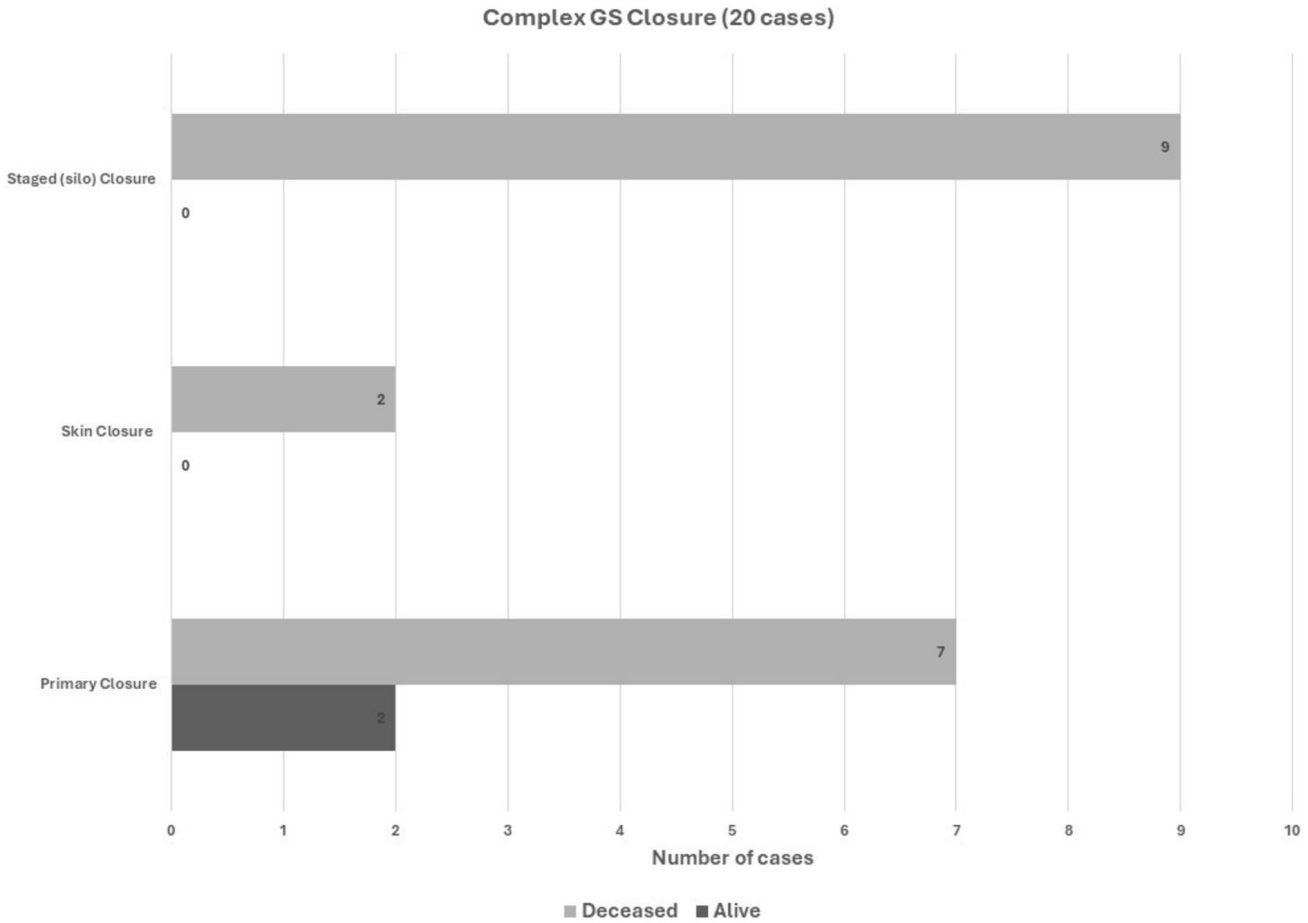
Surgical outcome in Complex GS

The survival rates in simple GS according to the type of surgery are summarized in Fig. 4. A primary closure of the abdominal wall had a significantly better survival compared to staged closure (51% vs 18%), P = 0.0001. Complex GS outcomes are similarly summarised in Fig. 5. Overall, simple GS had a significantly better survival than complex GS (40% vs 10%), P = 0.009. Survival among redo cases was 15% (3 of 20 cases).

## Discussion

To the best of our knowledge, this is the largest cohort of GS cases from Africa and the Middle East covering GS admissions from 2016 to 2024. This study represents a continuation of our ongoing effort to document and reflect the challenges confronting management of GS in Egypt. There was a slight female preponderance in keeping with our previous publication [21]. There is no specific gender incidence ratio in the literature, and it does not contribute to the risk factors [21, 23, 24]. Of interest, Räsänen et. al. found that being a male was an independent predictor of hospital stay duration [25]. Gestational age and birth weight were also within keeping with the literature [23, 24].

We approached GS survival with the following perspectives: overall survival, time to transfer, time to theatre and choice of surgery.

The survival trends in our cohort fluctuated throughout the years **(**Fig. 2**)**. There was a marginal improvement in survival from 2018 onwards compared to previous years however this did not reach statistical significance. The slight boost in survival may be attributed to a protocol-driven approach implemented on our unit since October 2017 [21]. The retrospective nature of the study precluded a detailed analysis of mortality. Nevertheless, it is clear that delayed transfer, the consequences of barotrauma, CLABSI and wound infection led to overwhelming sepsis in the majority of cases.

As expected, full-term neonates fared better than pre-term [24, 25] as did those with a normal birth weight compared to lower weights [26, 27].

The significantly better survival of cases transferred under 8 h is a key highlight of this study. A paucity in tertiary paediatric surgery centres and expertise as well as long transfer distances and dysfunctional referral networks are major contributors to poor outcomes in low resource settings [18, 21, 28–30].

Furthermore, a timely surgical intervention in less than 12 h has shown statistical significance compared to surgery thereafter. Numerous research investigations indicate that the interval between birth and surgical correction is essential in reducing mortality. Bilibio et al. found a higher mortality rate among GS patients who had surgery after more than 4 h of life [31].

We deplore the lack of a standardized national neonatal resuscitation and transport protocol. As all of our cases were outborn and transferred within the aforementioned time gaps, they all arrived with a varying degree of instability. Often cases would just “show up” at our Emergency Department, wrapped in cling film if available, gauze swabs, and oftentimes in a simple cloth. Transport and pre-surgical preparation are two critical factors in a LMIC setting and their optimization could directly influence survival rates.

In our cohort, complex GS patients had worse outcomes compared to simple GS, which is in concordance with the literature [9, 32]. Furthermore, primary closure cases fared better than staged surgical silo cases. Putting this finding in context with published literature is challenging as most studies consider the pre-formed, spring-loaded silo versus primary closure [32–34]. The studies conclude that there is no relationship between the method of closure and survival [32–34]. Chalresworth et al. includes the surgical silo in their cohort, however they group the latter with primary closure to compare them with pre-formed silo. Once more, there was no statistical significance between the groups regarding survival [35]. The inadequate outcomes of a phased closure strategy in our cohort can be related to additional risk factors such as sepsis [23, 24].

Finally, redo surgery is not uncommon in GS. The CAPS network reported that almost 25% of all GS patients underwent numerous surgeries throughout their hospitalization [36]. Friedmacher et al. showed that more than a quarter of their cases needed further surgeries [37]. GS patients with intestinal injury and other gastrointestinal abnormalities tend to have higher rates of morbidity and mortality [8, 23]. This study is limited by its retrospective nature where important data was difficult to obtain. An example of this are details relating to the causes of death. This would have been particularly advantageous since sepsis is largely preventable. Addressing infection control measures will greatly enhance survival.

## Conclusion

GS remains a challenging condition to manage in low and middle-income countries. Despite a slight improvement over the years, our survival rates are still below the international benchmark. Prompt and standardized resuscitation of GS cases is of crucial importance for survival. This study highlights the value of transferring GS cases to a paediatric surgery centre in under 8 h of birth. A primary fascial closure of the defect under 12 h of age appears to offer the best outcome.

## Data Availability

No datasets were generated or analysed during the current study.
